# Integrating routine blood biomarkers and artificial intelligence for supporting diagnosis of silicosis in engineered stone workers

**DOI:** 10.1002/btm2.10694

**Published:** 2024-06-28

**Authors:** Daniel Sanchez‐Morillo, Antonio León‐Jiménez, María Guerrero‐Chanivet, Gema Jiménez‐Gómez, Antonio Hidalgo‐Molina, Antonio Campos‐Caro

**Affiliations:** ^1^ Department of Engineering on Automation, Electronics and Computer Architecture and Networks University of Cádiz Cádiz Spain; ^2^ Biomedical Research and Innovation Institute of Cadiz (INiBICA) Cádiz Spain; ^3^ Pulmonology Department Puerta del Mar University Hospital Cádiz Spain; ^4^ Department of Analytical Chemical University of Cádiz Cádiz Spain; ^5^ Research Unit Puerta del Mar University Hospital Cádiz Spain; ^6^ Genetics Area, Biomedicine, Biotechnology and Public Health Department, School of Marine and Environmental Sciences University of Cadiz Cádiz Spain

**Keywords:** artificial stone, blood biomarkers, engineered stone, machine learning, silica agglomerate, silicosis

## Abstract

Engineered stone silicosis (ESS), primarily caused by inhaling respirable crystalline silica, poses a significant occupational health risk globally. ESS has no effective treatment and presents a rapid progression from simple silicosis (SS) to progressive massive fibrosis (PMF), with respiratory failure and death. Despite the use of diagnostic methods like chest x‐rays and high‐resolution computed tomography, early detection of silicosis remains challenging. Since routine blood tests have shown promise in detecting inflammatory markers associated with the disease, this study aims to assess whether routine blood biomarkers, coupled with machine learning techniques, can effectively differentiate between healthy individuals, subjects with SS, and PMF. To this end, 107 men diagnosed with silicosis, ex‐workers in the engineered stone (ES) sector, and 22 healthy male volunteers as controls not exposed to ES dust were recruited. Twenty‐one primary biochemical markers derived from peripheral blood extraction were obtained retrospectively from clinical hospital records. Relief‐*F* features selection technique was applied, and the resulting subset of 11 biomarkers was used to build five machine learning models, demonstrating high performance with sensitivities and specificities in the best case greater than 82% and 89%, respectively. The percentage of lymphocytes, the angiotensin‐converting enzyme, and lactate dehydrogenase indexes were revealed, among others, as blood biomarkers with significant cumulative importance for the machine learning models. Our study reveals that these biomarkers could detect a chronic inflammatory status and potentially serve as a supportive tool for the diagnosis, monitoring, and early detection of the progression of silicosis.

**Translational Impact Statement**

Engineered stone silicosis, caused by inhaling crystalline silica, presents a severe occupational health threat globally. Progressing rapidly from simple silicosis (SS) to fatal progressive massive fibrosis (PMF), it lacks effective treatment. Early detection remains challenging, despite existing diagnostic methods. This study explores the use of routine blood biomarkers and machine learning to differentiate between healthy individuals, SS, and PMF patients. Analysis of 107 silicosis patients and 22 controls reveals promising results. Biomarkers like lymphocyte count, angiotensin‐converting enzyme, and lactate dehydrogenase show significant potential for diagnosing and monitoring silicosis progression.

## 1 INTRODUCTION

Silica inhalation is one of the leading causes of occupational respiratory disease worldwide.^1^ Exposure to respirable crystalline silica (RCS) can cause silicosis, which can ultimately lead to progressive massive fibrosis (PMF), respiratory failure, and death. Silicosis is considered the most lethal and enduring occupational disease of the 20th century.^2^

Engineered stone (ES), also known as artificial stone (AS), is a new material that is growing in popularity in the construction industry as an alternative to natural stone.^3^ Silica agglomerates with high silica content are the earliest materials manufactured as ES and have become increasingly popular.^4^ They are resin‐based and have a silica content (quartz and cristobalite) greater than 80%.^5^ Although silicosis was considered to have a low prevalence in high‐income countries, it has experienced a resurgence in recent years. This resurgence is associated with the extensive use of ES in kitchen and bathroom countertops across numerous countries,^6^, ^7^, ^8^, ^9^ driven by the heightened demand for this material. Exposure to silica dust from ES during manufacturing, cutting, shaping, or polishing processes has significantly increased silicosis cases.^10^, ^11^

Epidemiological studies have highlighted a concerning trend of silicosis cases associated with the production and installation of AS surfaces. In the United States, it is estimated that over 100,000 workers are exposed to AS.^12^ In the last few years, the production and expansion of this product worldwide have not stopped growing, with an expected annual growth rate of 5.7%.^13^

Chest x‐rays (CXRs), high‐resolution computed tomography (HRCT), health and exposure questionnaires, and pulmonary function tests (PFT) are the main methods used in the diagnosis of silicosis in workers exposed to RCS.^14^, ^15^

Screening by CXRs is recommended by the International Labour Organization (ILO).^16^ The ILO system classifies lung parenchymal opacities by size, shape, and profusion by comparing chest radiographs with standard images. It is recommended that at least two readers classify radiographs independently because there is a considerable variation in classifying ILO CXRs inter‐observer (reader to reader) and intra‐observer (the same reader).^16^ Also, this variability has been shown when conflicts of interest are evident and the probability of a positive or negative diagnosis is in part dependent on the hirer of B‐reader (employer or miner/clamant)^17^ and it is of crucial importance in the early stages of the disease, where small opacities can be misleading.

HRCT is considered more specific and sensitive than CXRs in the early detection of silicosis and the identification of PMF.^18^ The combined use of CXRs and HRCT can improve accuracy and reduce false negatives (FN), especially in the early stages of silicosis.^19^ Nevertheless, the convenience of using HRCT as a screening procedure has been the object of some controversy due to its high cost, low accessibility, and radiation exposure.^20^

As it can be ascertained, despite the techniques available in clinical practice, early and precise detection of silicosis remains a challenging goal.^21^ Therefore, finding new, cost‐effective tools that aid in the precise diagnosis of the disease will be crucial.

Routine blood analysis has been proposed as a cost‐effective and easily measurable technique for improving the diagnosis and prognosis of various pathologies,^22^ such as some interstitial lung diseases, idiopathic pulmonary fibrosis,^23^, ^24^ or hypersensitivity pneumonitis.^25^ Furthermore, some recent studies show that certain biomarkers obtained from routine blood tests, such as lactate dehydrogenase (LDH), angiotensin‐converting enzyme (ACE), fibrinogen, and various cellular ratios, which are related to the chronic inflammatory state found in these patients even years after exposure cessation, could aid in distinguishing patients with simple silicosis (SS) from healthy subjects.^26^ These biomarkers could serve as an additional element to the diagnosis performed through imaging techniques, although the possible role of these biomarkers is not currently well known.

The rapid advancement of artificial intelligence (AI) technology provides an opportunity for its integration into clinical practice, potentially transforming healthcare services.^27^ The integration of AI in clinical applications serves as a tool to enhance disease diagnosis, reduce costs and time, and increase efficiency, ultimately resulting in improved patient care.^28^ When predicting health outcomes, conventional statistical analysis has been traditionally applied by calculating scores for risk stratification. The classic assumption is that there are a small number of important variables on which the model can be based. However, these variables often have a hidden interaction with each other. Machine learning, a subfield of AI, can play an efficient role in imaging, risk analysis, health information management, and early diagnosis by its ability to capture complex multivariate relationships and interactions between healthcare data that may be difficult for humans to interpret.^29^ In particular, the utilization of AI in analyzing blood test biomarkers has proven highly effective, revealing hidden patterns and providing valuable insights beyond the recognition of even experienced clinicians.^30^ Recent studies have proven an association between specific biomarkers identified in routine blood tests and a spectrum of diseases, such as autism,^31^ various types of cancer^30^, ^32^, ^33^ and the diagnosis of coronavirus disease 2019 (COVID‐19).^34^, ^35^

As far as we know, despite the benefits of AI in healthcare along with the potential of specific biomarkers from routine blood tests and their easy accessibility, the combined use of routine indices and AI in ES silicosis patients remains unexplored. Accordingly, this study aimed to determine whether biomarkers from routine blood tests can be used together with AI techniques to discriminate between healthy, patients diagnosed with SS, and patients with PMF, providing a clinical decision support tool that can complement existing modalities for the diagnosis and monitoring of silicosis.

## 2 MATERIALS AND METHODS

### 2.1 Participants

A total of 129 subjects were selected and agreed to participate in this study. Data were obtained retrospectively from clinical hospital records (2009–2016) and prospectively from 2017 until 2023. Patients with silicosis (*N* = 107) were males with a history of working in cutting, polishing, and finishing ES countertops. They were all followed by the Pneumology Department of Puerta del Mar University Hospital in Cádiz (Spain). The diagnosis was based on a history of silica exposure and chest radiography and/or HRCT and, in some cases, lung or mediastinal lymph node biopsy. Radiological findings were characterized as per the ILO system^16^ and the International Classification of HRCT for Occupational and Environmental Respiratory Diseases.^36^, ^37^

All patients ceased their exposure at diagnosis. In addition, male volunteers (*N* = 22) with no history of RCS exposure were used as the healthy control (HC) group. They were hospital staff workers without respiratory symptoms or chronic/acute disease.

The research followed the guidelines of the Declaration of Helsinki and received approval from the Research Ethics Committee of the province of Cadiz. Informed consent was obtained from all study participants, which allowed access to the data collected from blood routine tests conducted during the diagnosis and follow‐up of the disease from 2009 to 2023.

### 2.2 Characteristics of the study subjects and respiratory clinical data

Demographic and clinical data of the participants were collected from the patient's clinical history or by face‐to‐face interview during their medical appointment. Data included starting exposure age, diagnosis age, and duration of exposure to ES in years.

Respiratory function tests were performed by trained personnel using a Master Screen PFT/Body System (Jaeger‐Viasys, CareFusion, or EasyOne Pro, ndd Medizintechnik AG) on the same day of blood extraction. The data collected included forced expiratory volume in 1 s (FEV1), forced vital capacity (FVC), the ratio of FEV1/FVC, and the diffusing capacity of the lung for carbon monoxide (DLCO), estimated following the single‐breath procedure according to international recommendations.

### 2.3 Biochemical and hematological blood markers

To identify differences among the three examined groups, we assessed the primary biochemical markers derived from peripheral blood extraction. Overnight‐fasting blood samples were collected into ethylenediaminetetraacetic acid (EDTA) tubes and promptly processed for general hematological and biochemistry analysis in the Clinical Analysis Department at the Puerta del Mar University Hospital. In total, 21 biomarkers were quantified, including enzymatic biochemical parameters, white blood cell (WBC) counts and percentage subsets, and WBC‐derived ratios. More specifically, three enzymatic biochemical parameters were measured, namely alkaline phosphatase (ALP) in units per liter (U/L), LDH in units per milliliter (U/mL), and the ACE in units per liter (U/L). Concerning the WBC subsets, the total leukocyte numbers and the subsets including neutrophils, eosinophils, basophils, monocytes, lymphocytes, and platelets were counted. Monocytes, eosinophils, basophils, lymphocytes, and neutrophils percentages were also estimated. Finally, WBC ratios were calculated, including the platelet/lymphocyte ratio (PLR), the neutrophil/lymphocyte ratio (NLR), the lymphocyte‐to‐monocyte ratio (LMR), the systemic immune‐inflammation index (SII) (neutrophil × platelet/lymphocyte ratio), the systemic inflammation response index (SIRI) (neutrophil × monocyte/lymphocyte ratio), and the aggregate index of systemic inflammation (AISI) (neutrophil × monocyte × platelet/lymphocyte ratio).

### 2.4 Data processing and statistics

#### 2.4.1 Data set

The 107 patients with silicosis were periodically followed from 2009 to 2023. During the study, a total of 680 visits were recorded, with an average of six visits per patient, resulting in 680 different data samples, which included 22 samples of HC subjects, 441 samples of patients with SS, and 217 samples corresponding to patients with PMF. Data samples were pooled. The label associated with each sample (HC, SS, and PMF) for model training corresponded to the diagnosis at the time of the blood test.

#### 2.4.2 Data oversampling

The class imbalance problem in machine learning refers to the scenario where there is a disproportionate distribution of samples among different classes, leading to a bias in learning toward the majority classes. To address the imbalanced subsets of HC and PMF subjects, new samples were synthesized from the samples. For this data oversampling procedure, the Synthetic Minority Oversampling Technique (SMOTE) was applied.^38^ SMOTE operates by selecting the *k*‐nearest minority class neighbors that are close in the space of features and then uses linear interpolation to generate new samples. This tabular data approach has gained popularity as one of the most effective solutions to tackle class imbalance problems in machine learning tasks.

#### 2.4.3 Missing data imputation and feature selection

A mean imputation strategy for each patient group was used for missing values.^39^ In addition, feature selection was used to reduce the dimensionality of the data set by identifying the biomarkers with the highest discriminating capacity.^40^ The rank importance of biomarkers was assessed using the Relief‐F algorithm.^41^ This feature selection algorithm calculates the weights for each feature based on the distance between samples and their nearest neighbors, generating a variable ranking based on these weights. Weights range from −1 to 1. The larger the positive weight, the greater the importance of the predictor.

#### 2.4.4 Machine learning models

We explored the potential of machine learning techniques to classify blood samples from HC, SS, and PMF patients. Five machine learning models were trained and validated using the selected biomarkers.

##### Support vector machines

Support vector machines (SVM) models are supervised machine learning methods that classify data samples by finding the appropriate hyperplane that best separates classes in a high‐dimensional space.^42^ Recently, SVM has been used in the respiratory field with blood biomarkers for predicting early COVID‐19 mortality^43^ and for exploring how immune cell infiltration contributes to chronic obstructive pulmonary disease (COPD); pathogenesis.^44^ In this study, a radial‐basis kernel and a grid search for optimal parameters with a four‐fold cross‐validation (CV) were used for training the SVM classifier.

##### Probabilistic neural network

A probabilistic neural network (PNN) is a supervised machine learning model that implements the kernel discriminant analysis algorithm by structuring operations within a multilayered feed‐forward network.^45^ PNN is a neural network model that computes posterior probabilities by combining outputs from individual neurons using a probabilistic framework. The network architecture presents four fully interconnected layers. The input layer has as many neurons as the number of features in the training set. The output layer bases the decision on the Bayes optimal decision rule. In respiratory medicine, PNNs have been extensively explored, with recent applications to asthma screening,^46^ and lung cancer classification using x‐ray images.^47^ In this study, the PNN classifier was trained with a sigma search control, and unnecessary neurons were removed to minimize the error.

##### Linear discriminant

Linear discriminant (LD) is a classification technique that generalizes the Fisher LD and finds the best linear combination of features to separate classes. LDA is a well‐known machine‐learning algorithm, which has been broadly used in the respiratory medicine domain. Very recently, LDA was used to predict overall survival and response to immunotherapy in non‐small cell lung cancer,^48^ and to discriminate the breathprints of healthy from COPD subjects.^49^

##### *k*‐Nearest neighbors

*k*‐Nearest neighbors (KNN) is a non‐parametric supervised learning algorithm used for classification. It determines the class of new data by examining the majority class among their *k* nearest neighbors. The input for the KNN algorithm involves the *k‐*closest training instances in the training data set. In recent years, KNN has been used in the diagnosis of silicosis with an electronic nose^50^ and in the early lung tumor prediction based on metabolomic biomarkers.^51^ In this work, *k* values in the range from 2 to 100 were explored, using a four‐fold CV strategy.

##### Decision tree

A decision tree (DT) is a hierarchical supervised model represented as a tree where nodes are split into sub‐nodes based on a threshold value of an attribute. Decisions are made at each node based on feature values, leading to the final prediction at the leaf nodes. The DT learns simple decision rules deduced from the training data. In this study, the DT model was built considering five as the minimum number of rows in a node and 10 as the maximum tree levels. The tree pruning to minimize error was performed using 10‐fold cross‐validation. DTs have been used, for example, to identify lung cancer diagnosis using empirical negative control microRNAs,^51^ and for the screening of serum biomarkers of silicosis.^52^

#### 2.4.5 Performance metrics and validation

The following performance metrics, commonly used to assess the performance of machine learning models, were calculated for all classifiers: accuracy (Acc), sensitivity (Se), and specificity (Sp), the geometric mean of sensitivity and specificity (Gmean), positive (PPV), and negative (NPV) predictive values rates, recall, *F*‐measure, and precision (Pr).

CV was used to estimate the predictive performance of models. CV operates by fitting each model to a subset of the available data (training set). Then the models' predictive features are estimated on the remaining subset of the data (test set). This procedure is iterated by selecting different subsets of data. The overall predictive metrics are averaged across iterations. In this study, 10 iterations were used (10‐fold CV). The performance metrics were calculated using the 3‐classes confusion matrix illustrated in Table 1. Each column represents the instances of an actual class, while each row represents the instances of a predicted class. The confusion matrix provides information on the number and type of errors made by a classifier. The number of true positive (TP) samples in class HC is TP_HC_, that is, the number of samples that are correctly classified from class HC; and *E*_HC‐SS_ is the number of samples from class HC that were incorrectly classified as class SS, that is, misclassified samples. Thus, the number of FN in the HC class (FN_HC_) is the sum of all class HC samples that were incorrectly classified as class SS or PMF: FN_HC_ = *E*_HC‐SS_ + E_HC‐PMF_. Accordingly, the FN of any class that is in a column can be calculated by summing the errors in that class. The false‐positives (FP) for any predicted class that is in a row are obtained as the sum of all errors in that row. For example, the FP in class HC (FP_HC_) is calculated as FP_HC_ = *E*_SS‐HC_ + *E*_PMF‐HC_.^53^

**TABLE 1:** Confusion matrix. The green diagonal represents correct predictions, and the blue cells indicate incorrect predictions.

	Actual HC	Actual SS	Actual PMF	
Predicted HC	TP_HC_	E_SS‐HC_	*E*_PMF‐HC_	
Predicted SS	*E*_HC‐SS_	TP_SS_	*E*_PMF‐SS_	
Predicted PMF	*E*_HC‐PMF_	*E*_SS‐PMF_	TP_PMF_	

Abbreviations: HC, healthy subjects; PMF, pulmonary massive fibrosis; SS, simple silicosis; E, incorrectly classified; TP, true positive.

Equations (1)–(8) provide an overview of the metrics defined for each specific class *k*.

(1) $${\mathrm{Acc}}_{k}=\frac{TP_{k}}{TP_{k}+TN_{k}+FP_{k}+FN_{k}}.$$

(2) $${\mathrm{Se}}_{k}=\frac{{\mathrm{TP}}_{k}}{TN_{k}+FP_{k}}.$$

(3) $${\mathrm{Sp}}_{k}=\frac{{\mathrm{TN}}_{k}}{TN_{k}+FP_{k}}.$$

(4) $${\text{Gmean}}_{k}=\sqrt{Se_{k}Sp_{k}}.$$

(5) $${\mathrm{PPV}}_{k}=\frac{{\mathrm{TP}}_{k}}{{\mathrm{FP}}_{k}+{\mathrm{FP}}_{k}}.$$

(6) $${\mathrm{NPV}}_{k}=\frac{{\mathrm{TN}}_{k}}{{\mathrm{TN}}_{k}+{\mathrm{FN}}_{k}}.$$

(7) $${\text{Recall}}_{k}=\frac{{\mathrm{TP}}_{k}}{{\mathrm{TP}}_{k}+{\mathrm{FN}}_{k}}.$$

(8) $$F1\;{\text{Score}}_{k}=\frac{2{\mathrm{TP}}_{k}}{2{\mathrm{TP}}_{k}+{\mathrm{FP}}_{k}+{\mathrm{FN}}_{k}}.$$

(9) $$\mathrm{Pr}=\frac{{\mathrm{TP}}_{k}}{{\mathrm{TP}}_{k}+{\mathrm{FP}}_{k}}.$$

The importance of variables in a machine learning model plays a crucial role in its explainability. By understanding which variables are most influential in predicting the outcome, a deeper understanding of how the model operates and which features of the data set contribute significantly to the model's decisions can be gained. In this study, the biomarker's importance was computed during the CV procedure. Importance was estimated for each CV fold. The geometric mean of the importance across folds was calculated as a way to compute variable importance that minimizes the importance of variables that are relatively unimportant on any CV fold.

#### 2.4.6 Statistical analysis

Statgraphics 19 (Statgraphics‐Technologies, Inc., The Plains, VA, USA) and Matlab R2023b (Mathworks Inc., Natick, MA) were used for statistical analysis. GraphPad Prism 10 (Prism Software Corp., Irvine, CA, USA) was used for graphical representations. DTREG (Sherrod, P. Brentwood, TN, USA) was used for feature selection and building the machine learning models.

## 3 RESULTS

### 3.1 Participants

The sociodemographic and pulmonary function data of the 107 patients with silicosis (SS and PMF) at the time of the initial diagnosis are shown in Table 2, including age at the start of work as an ES handler, age at diagnosis, duration of exposure to ES, and lung functional parameters. The starting exposure age, diagnosis age, and duration of exposure to RCS were all similar, with no significant differences between the SS and PMF groups.

**TABLE 2:** Sociodemographic data and pulmonary function values of patients with simple silicosis (SS) and pulmonary massive fibrosis (PMF) at the time of the initial diagnosis. Data are mean value ± standard deviation. ANOVA: values in the same row with different letters indicate significant differences (*p* < 0.05).

	SS (*n* = 100)	PMF (*n* = 7)	*F*‐statistics	*p*‐Value
Starting exposure age (years)	21.2 ± 6.2	21.6 ± 3.3	1.5	0.224
Diagnosis age (years)	36.7 ± 7.3	38.1 ± 6.9	0.54	0.464
Duration of exposure (years)	13.3 ± 6.3	12.4 ± 6.0	1.5	0.224
FEV_1_ (mL)	3381.4 ± 683.9^b^	3000.2 ± 659.8^a^	70.51	<0.0001
FEV_1_ (%)	87.1 ± 13.9^b^	75.1 ± 16.5^a^	104.31	<0.0001
FVC (mL)	4320.4 ± 815.9^b^	3971.3 ± 811.0^a^	25.59	<0.0001
FVC (%)	87.9 ± 14.3^b^	82.1 ± 16.2^a^	24.46	<0.0001
FEV1/FVC	0.8 ± 0.1^b^	0.7 ± 0.1^a^	117.58	<0.0001
DLCO (mmol/min/kPa)	9.0 ± 1.9^b^	8.3 ± 1.8^a^	16.58	0.0001
DLCO (%)	84.7 ± 19.1^b^	78.9 ± 17.2^a^	12.36	0.0005

Abbreviations: DLCO, diffusing capacity of the lung for carbon monoxide; FEV_1_, forced expiratory volume in 1 s; FVC, forced vital capacity.

Significant differences were observed in the mean values for FEV_1_, FVC, DLCO, and FEV_1_/FVC between the SS and PMF groups. All average values related to respiratory functions were lower in the group of patients diagnosed with PMF compared to those diagnosed with SS. At the beginning of the study, 100 out of the 107 patients with silicosis had a diagnosis of SS and 7 of PMF. In the SS group, 42% (*n* = 42) progressed to PMF throughout the study, with the mean progression period being 4.9 ± 2.2 years from the first diagnosis. At the onset of the study, 17% (*n* = 18) of patients were active smokers, 43% (*n* = 46) were former smokers, and 40% (*n* = 43) were non‐smokers.

### 3.2 Data augmentation

SMOTE was applied for synthesizing new observations of minority classes HC and PMF. In this study, *k* = 1 and *k* = 19 values were used to augment the number of samples in the PMF and HC subsets, respectively, resulting in a final data set of 1323 data samples according to the following distribution: 441 HC, 441 SS, and 441 PMF. Samples were randomly shuffled. The dimensions of the resulting data matrix were 1323 × 21.

### 3.3 Features selection

As detailed in Section 2.3, the parameters considered in the blood routine tests for this study included: neutrophils percentage, lymphocytes percentage, monocytes percentage, eosinophils percentage, basophiles percentage, leukocytes count, basophils count, neutrophils count, lymphocytes count, monocytes count, eosinophils count, NLR, platelets, SII, LMR, PLR, SIRI, AISI, ALP, LDH, ACE.

**FIGURE 1:**
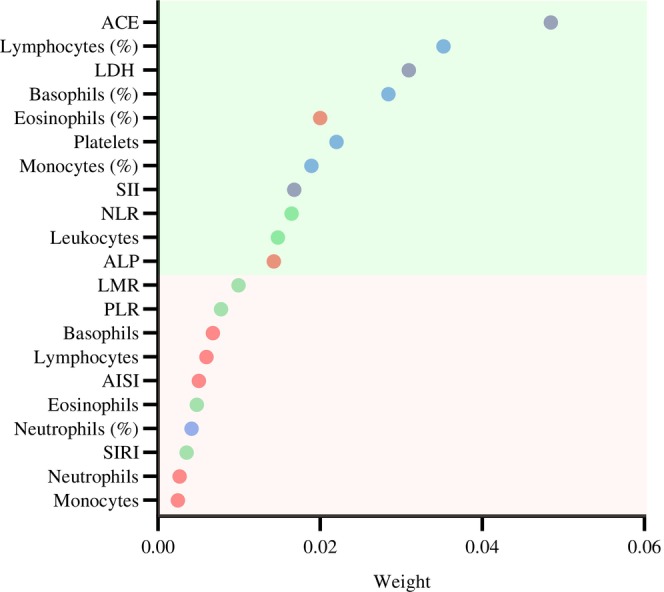
Feature ranking by Relief‐*F* algorithm. Relief‐*F* weights were applied to rank and select top‐scoring features for feature selection. The biomarkers with weights >0.01 were selected to build the machine learning models. ACE, angiotensin‐converting enzyme; AISI, aggregate index of systemic inflammation; ALP, alkaline phosphatase; LDH, lactate dehydrogenase; LMR, lymphocyte‐to‐monocyte ratio; NLR, neutrophil/lymphocyte ratio; PLR, platelet/lymphocyte ratio; SII, systemic immune‐inflammation index; SIRI, systemic inflammation response index.

The Relief‐*F* algorithm, able to deal with multiclass problems, was applied to estimate the quality of the features and rank them based on their discriminative capacity. Relief‐*F* provides as an output a vector of estimations of the qualities of biomarkers. The resulting feature ranking is shown in Figure 1.

Variables were ordered from highest to lowest relevance in discriminating between groups. The features with Relief‐*F* weights greater than 0.01 were selected, namely: ACE, lymphocytes percentage, LDH, basophils percentage, eosinophils percentage, platelets count, monocytes percentage, SII, NLR, leukocytes count, and ALP. This procedure allowed to reduce the dimensionality of the working matrix to 1323 × 11.

**FIGURE 2:**
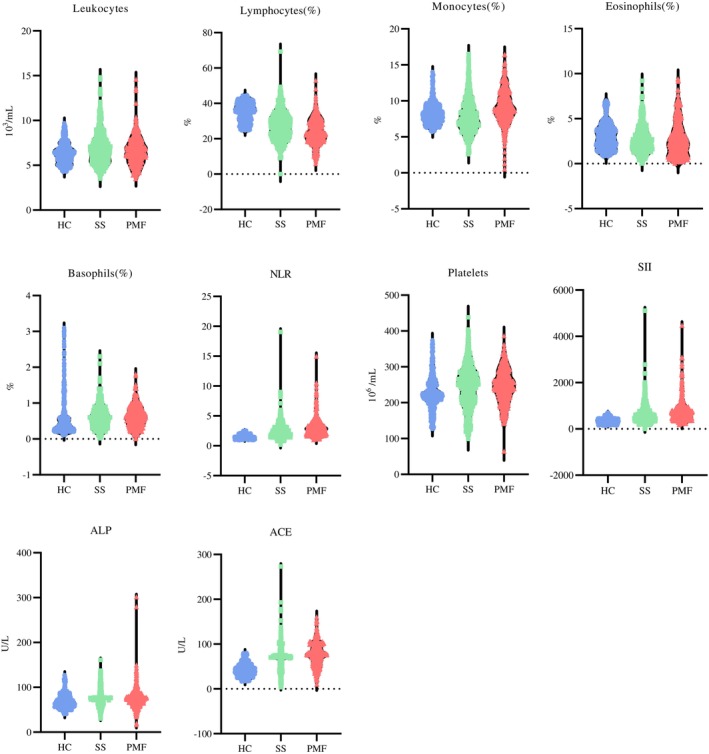
Violin plots showing results from blood test selected markers. ACE, angiotensin‐converting enzyme; ALP, alkaline phosphatase; HC, healthy subjects; NLR, neutrophil/lymphocyte ratio; PMF, pulmonary massive fibrosis; SII, systemic immune‐inflammation index; SS, simple silicosis.

Table 3 details the statistical information of the selected biomarkers. Figure 2 shows the violin plots of the reduced subset of blood markers.

**TABLE 3:** Data are mean value ± standard deviation. ANOVA: homogenous groups are identified using columns of letters. Within each column, the levels containing the same letter form a group of means within which there are no statistically significant differences (*p* > 0.05).

	HC	SS	PMF
ACE	43.13 ± 13.29^a^	70.08 ± 27.22^b^	76.43 ± 24.60^c^
Lymphocytes (%)	34.79 ± 6.58^a^	27.25 ± 7.94^b^	23.97 ± 7.40^c^
LDH	191.05 ± 47.29^a^	217.06 ± 55.73^b^	262.06 ± 67.77^c^
Basophils (%)	0.59 ± 0.68	0.62 ± 0.34	0.62 ± 0.31
Eosinophils (%)	3.10 ± 1.79	2.67 ± 1.48	2.70 ± 2.06
Platelets	228.67 ± 56.35	244.74 ± 58.76	244.10 ± 48.85
Monocytes (%)	8.34 ± 1.87^ab^	7.88 ± 2.38^a^	9.22 ± 2.37^b^
SII	355.13 ± 141.00^a^	629.75 ± 419.44^b^	762.88 ± 506.15^c^
NLR	1.56 ± 0.45^a^	2.57 ± 1.45^b^	3.08 ± 1.74^c^
Leukocytes	6.35 ± 1.42^ab^	6.87 ± 1.76^a^	6.55 ± 1.58^b^
ALP	68.68 ± 19.80	76.13 ± 17.70	75.66 ± 24.63

Abbreviations: ACE, angiotensin‐converting enzyme; ALP, alkaline phosphatase; HC, healthy subjects; LDH, lactate dehydrogenase; PMF, pulmonary massive fibrosis; NLR, neutrophil/lymphocyte ratio; SII, systemic immune‐inflammation index; SS, simple silicosis; TP, true positive.

The count of leukocytes, the percentage of lymphocytes and monocytes, the NLR, and the SII, LDH, and ACE indexes reveal significant differences among the groups. With the percentage of lymphocytes, higher values were found in the HC control group. Values of this marker in the SS group were greater than in the PMF group. The NLR behaved inversely, with uniformly increasing values, from the group of healthy subjects to the PMF group. The SII, LDH, and ACE increase markedly with the onset of SS, being higher in the group of subjects with PMF.

As for the leukocyte and platelet counts and the ALP index, the mean values in patients with silicosis are higher than those in the group of patients without the disease.

### 3.4 Machine learning models for sample identification

**FIGURE 3:**
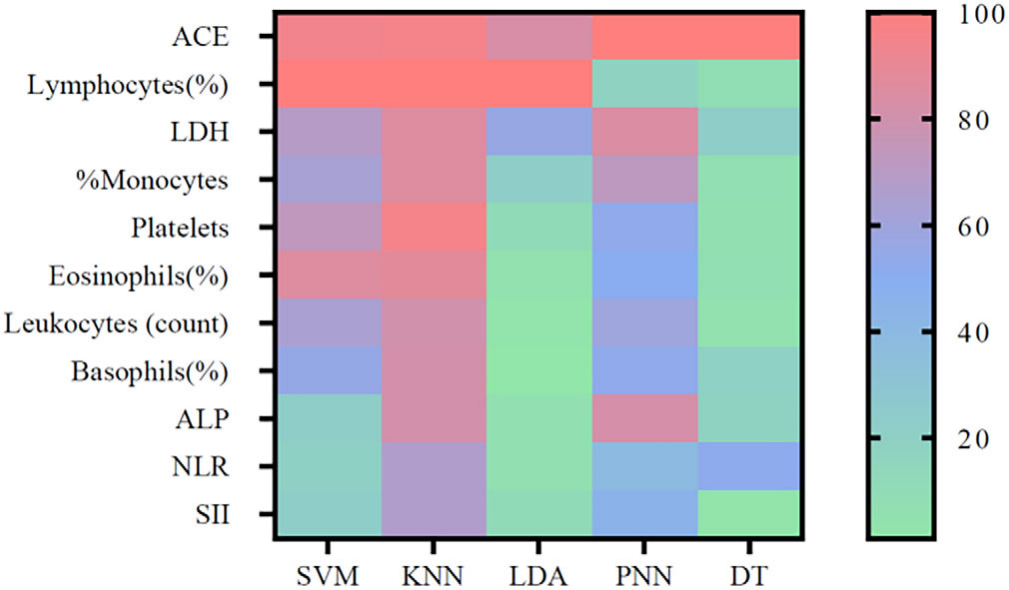
Importance of blood biomarkers in classification models. ACE, angiotensin‐converting enzyme; ALP, alkaline phosphatase; DT, decision tree classifier; KNN, *k*‐nearest neighbors; LDA, linear discriminant analysis; LDH, lactate dehydrogenase; NLR, neutrophil/lymphocyte ratio; PNN, probabilistic neural network; SII, systemic immune‐inflammation index; SVM, support vector machines.

Five classification algorithms (SVM with radial kernel, PNN, LD, KNN, and DT) were trained and validated using the selected biomarkers identified by the proposed feature selection approach. Table 4 details the evaluation scores of each classification algorithm, classified by group of subjects. Table 5 presents the weighted metrics for each model. In addition, Figure 3 shows the importance of blood biomarkers in the classification models. The importance of each variable for each model ranges from 0% to 100%.

**TABLE 4:** Performance metrics of the machine learning models over the validation set.

Group	Model	Acc	Se	Sp	Gmean	PPV	NPV	Pr	Recall	*F*‐measure
HC	SVM	0.98	0.98	0.98	0.98	0.96	0.99	0.96	0.98	0.97
	KNN	0.94	0.99	0.91	0.95	0.85	1.00	0.85	0.99	0.91
	LDA	0.87	0.88	0.87	0.87	0.76	0.93	0.76	0.88	0.82
	PNN	0.96	0.99	0.94	0.97	0.89	1.00	0.89	0.99	0.94
	DT	0.93	0.94	0.93	0.93	0.87	0.97	0.87	0.94	0.90
SS	SVM	0.87	0.80	0.91	0.85	0.82	0.90	0.82	0.80	0.81
	KNN	0.84	0.63	0.94	0.77	0.84	0.84	0.84	0.63	0.72
	LDA	0.75	0.58	0.84	0.69	0.64	0.80	0.64	0.58	0.60
	PNN	0.87	0.70	0.95	0.82	0.89	0.87	0.89	0.70	0.78
	DT	0.83	0.70	0.90	0.80	0.78	0.86	0.78	0.70	0.74
PMF	SVM	0.89	0.83	0.92	0.87	0.83	0.92	0.83	0.83	0.83
	KNN	0.87	0.85	0.89	0.87	0.79	0.92	0.79	0.85	0.82
	LDA	0.82	0.71	0.88	0.79	0.74	0.86	0.74	0.71	0.73
	PNN	0.90	0.89	0.90	0.90	0.82	0.94	0.82	0.89	0.85
	DT	0.86	0.80	0.89	0.84	0.78	0.90	0.78	0.80	0.79

Abbreviations: Acc, accuracy; DT, decision tree classifier; Gmean, geometric mean of specificity and sensitivity; HC, healthy; KNN, *k*‐nearest neighbors; LDA, linear discriminant analysis; PMF, pulmonary massive fibrosis; PNN, probabilistic neural network; PPV, positive predictive value; Pr, precision; Se, sensitivity; Sp, specificity; SS, simple silicosis; SVM, support vector machines.

**TABLE 5:** Weighted performance metrics of the machine learning models over the validation set.

Model	Acc	Se	Sp	Gmean	PPV	NPV	Pr	Recall	*F*‐measure
SVM	0.91	0.87	0.93	0.9	0.87	0.93	0.87	0.87	0.87
PNN	0.91	0.86	0.93	0.89	0.86	0.93	0.86	0.86	0.86
KNN	0.88	0.82	0.91	0.86	0.82	0.92	0.82	0.82	0.82
DT	0.88	0.81	0.91	0.86	0.81	0.91	0.81	0.81	0.81
LDA	0.81	0.72	0.86	0.78	0.72	0.86	0.72	0.72	0.72

Abbreviations: Acc, accuracy; DT, decision tree classifier; Gmean, geometric mean of specificity and sensitivity; HC, healthy; KNN, *k*‐nearest neighbors; LDA, linear discriminant analysis; PMF, pulmonary massive fibrosis; PNN, probabilistic neural network; PPV, positive predictive value; Pr, precision; Se, sensitivity; Sp, specificity; SS, simple silicosis; SVM, support vector machines.

## 4 DISCUSSION

Despite the advanced medical imaging techniques available in clinical practice today, the early detection of silicosis caused by ES remains a challenge. Routine blood testing has been shown to be a cost‐effective and accessible technique in clinical settings. Biomarkers derived from blood tests can be used in conjunction with AI techniques to exploit patterns of interrelationship, which are often hidden in conventional statistical analyses, such as in the case of univariate methods.

A cohort of 107 silicosis patients has been followed up during the period from 2009 to 2023. During this period, routine blood tests were performed periodically, obtaining a longitudinal follow‐up of 21 blood biomarkers linked to the diagnosis of each patient.

These biomarkers were analyzed and used in conjunction with machine learning techniques to attempt to automatically discriminate healthy subjects from those affected by silicosis in its initial (simple silicosis) and advanced (PMF) stages. Feature selection techniques allowed for a reduction of the initial set of biomarkers to subsequently train and validate various machine learning models.

The methodology applied has allowed the identification of a new subset of biomarkers with potential for the diagnosis, including the ACE, LDH, ALP, and SII indexes; the percentage of lymphocytes, basophils, eosinophils, and monocytes; the count of platelets and leukocytes; and the NLR.

The resulting models have shown a significant performance, allowing a correct classification between the studied groups (HC, SS, and PMF) with remarkable accuracy. As Table 4 shows, sensitivities greater than 82% and specificities greater than 89% were achieved in the best cases. All models had a high weighted accuracy (>0.8). In the HC group, the SVM gave the best accuracy (0.98), sensitivity (0.97), and specificity (0.98). In the SS and PMF groups, the SVM offered again the best diagnosis accuracy (0.87 and 0.89, respectively), sensitivity (0.84 and 0.82, respectively), and specificity (0.89 and 0.93, respectively).

As can be observed in Figure 3, the percentage of lymphocytes and the ACE and LDH indexes were the blood biomarkers with higher cumulative importance for the machine learning models. Most of the retained biomarkers are related to a chronic inflammatory state. Scientific literature supports a connection between some of the selected biomarkers and silicosis, which confirms their discriminative potential.^26^, ^54^ Silicosis patients are in a chronic inflammatory state induced by silica. Inhaling crystalline silica dust results in the accumulation of these particles within the lungs, where alveolar macrophages are unable to clear them, triggering an inflammatory reaction that culminates in lung fibrosis. Prior research has established a correlation between elevated levels of inflammatory markers and increased susceptibility to silicosis, heightened radiological severity, and diminished pulmonary function among those exposed to silica.^26^, ^55^, ^56^, ^57^

The WBCs are associated with the diagnosis of certain diseases and are involved in the body's immune response. These cells could serve as indicators of inflammatory state and severity, aiding in the diagnosis and clinical prognosis of patients with ES silicosis, as demonstrated in recent studies.^26^ They might reflect the progression and stage of patients. Recent studies have shown lower lymphocyte counts in individuals with silicosis compared to unexposed and exposed individuals without silicosis, as is the case in our study.^56^

The elevation of ACE in the serum of these patients may originate from immune cells or endothelial cells and could be directly associated with lung tissue damage.^58^, ^59^ Additionally, several researchers have verified that serum ACE activity is elevated in a significant portion of patients with granulomatous diseases such as sarcoidosis and silicosis.^55^, ^57^

Platelets also play a significant role, beyond hemostasis, coagulation, and angiogenesis, in innate immunity and the inflammatory response. They are involved as mediators in the body's inflammatory response, releasing inflammation mediators. Although cohorts of patients from other studies have not shown higher levels of this biomarker,^56^ it has proven to be highly relevant in our study. The mentioned work did not address the relevance of these biomarkers using a multivariate strategy.

ALP is used to diagnose and monitor various health conditions and is also linked to the body's inflammatory state. Although no studies have found ALP in routine blood tests as a potential biomarker for diseases such as silicosis, studies in the literature have found a high presence of this enzyme in lung fluids from patients with silicosis.^60^ The greater the progression of the pulmonary disease with interstitial fibrosis, the greater the presence of ALP in the fluid. ALP reflects fibrosing evolution.

While serum LDH is a nonspecific marker of cell damage, numerous studies in both human and animal models have suggested its utility as a marker for silicosis.^61^, ^62^ The elevation in plasma LDH concentration can also be observed in various other conditions, including systemic infections or inflammations, muscle injuries, hemolysis, thromboembolism, or malignancies. LDH levels increase in response to inflammatory and fibrotic processes in the lungs. Previous research has shown a significant rise in plasma LDH levels among individuals with prolonged silica exposure and those diagnosed with silicosis compared to unexposed individuals.^26^, ^55^, ^56^

In our study, a multivariate approach is proposed. Biomarkers that may not appear to be relevant a priori for the classification of patients have shown to be so since the machine learning techniques used can discover hidden patterns between the parameters. It is usual to find cases in which statistically significant predictors do not become useful predictors in multivariate models.^63^ It is the case of the SII and NLR indexes, which showed statistical significance for discriminating groups but accounted for a lower predictive capacity in the validated machine learning models. On the other hand, it can happen that variables with strong predictivity sometimes fail to be significant.^64^ In this study, it happens to eosinophils and basophils, which have not been described as biomarkers of silicosis previously, but in some of the machine learning models, such as SVM and KNN, have a relevant role in the classification task.

Although chest radiography is the initial and most commonly used diagnostic test, a significant percentage of cases present with normal CXRs. Nineteen of 106 patients (18%) in the series published by León‐Jiménez et al.,^15^ 36 of 117 (40%) in the series by Hoy et al.,^65^ 43% of 78 patients in the series by Newbigin et al.^66^ or 37% in the series by Orriols et al.^67^ were classified as ILO category 0. On the other hand, a biopsy was required for diagnosis in 34 of 52 (65%) patients in the series of Fazio et al.,^9^ as well as in 14%^15^ and 17%^67^ in two of the studies abovementioned. These data support the complexity of diagnosing silicosis due to AS. The use of these biomarkers could aid in diagnosis, especially in the initial disease phases where radiology is inconclusive or in exposed workers, to indicate the need for HRCT in this subgroup of patients.

This study has some limitations. Firstly, a larger sample size is needed to corroborate the results and the robustness of the machine learning models. While the current data set has provided valuable insights, expanding the data set can enhance the model's predictive accuracy and reliability, enabling it to capture more effectively the full spectrum of variability and complexities inherent in this problem. Therefore, future work will prioritize the collection of additional data, including data from a group of exposed subjects without silicosis, to validate and strengthen the findings presented in this study.

In addition, the non‐specificity of the biomarkers can be affected by deviations in the health status of the patients attributable to conditions different from silicosis. Additionally, the progression of the disease is a continuous process that does not change abruptly from 1 day to the next. Therefore, there may be stages in the patient where the diagnosis between SS and PMF is not entirely clear, potentially causing some overlap between groups and high variability in the data.

Finally, the role of inflammatory indices may not only be limited to supporting diagnosis. The study of their changes along with the progression of the disease may open new research opportunities for a better understanding of the pathophysiology of the disease.

## 5 CONCLUSIONS

Eleven routine blood biomarkers were selected and used to train machine learning models for the discrimination between healthy subjects and patients with simple silicosis and PMF. This classification of patients based on biomarkers that could detect a chronic inflammatory status not only allows for discrimination between healthy and diseased patients, providing support for diagnosis, but also enables classification between different degrees of the disease (SS or PMF). These biomarkers can be obtained quickly and inexpensively since they are present in most blood test routines. These results could potentially serve as a supportive tool for the diagnosis, monitoring, and early detection of the progression of silicosis.

## CONFLICT OF INTEREST STATEMENT

The authors declare no conflict of interest.

## Data Availability

The data are not publicly available due to privacy or ethical restrictions. The data that support the findings of this study are available on request from the corresponding author for researchers who meet the criteria for confidential data access as stipulated by participant informed consent and the Institutional Research Ethics Committee of the province of Cadiz, Spain (register numbers 151.22, 90.18, 157/16‐SIL‐2016‐01, and 06.20).
